# Nuclear lymphocyte-specific protein tyrosine kinase and its interaction with CR6-interacting factor 1 promote the survival of human leukemic T cells

**DOI:** 10.3892/or.2015.3990

**Published:** 2015-05-19

**Authors:** SHAHROOZ VAHEDI, FU-YU CHUEH, SUJOY DUTTA, BALA CHANDRAN, CHAO-LAN YU

**Affiliations:** Department of Microbiology and Immunology, H.M. Bligh Cancer Research Laboratories, Chicago Medical School, Rosalind Franklin University of Medicine and Science, North Chicago, IL 60064, USA

**Keywords:** lymphocyte-specific protein tyrosine kinase, leukemia, CRIF1, non-canonical signaling, cell survival, proximity ligation assay

## Abstract

Overexpression and hyperactivation of lymphocyte-specific protein tyrosine kinase (Lck) have been associated with leukemia development. We previously showed that, other than its known function as a cytoplasmic signal transducer, Lck also acts as a nuclear transcription factor in mouse leukemic cells. In the present study, we demonstrated the presence of nuclear Lck in human leukemic T cells and in primary cells. We further established a positive correlation between Lck nuclear localization and its kinase activity. Proteomic analysis identified CR6-interacting factor 1 (CRIF1) as one of the Lck-interacting proteins. CRIF1 and Lck association in the nucleus was confirmed both by immunofluorescence microscopy and co-immunoprecipitation in human leukemic T cells. Close-range interaction between Lck and CRIF1 was validated by *in situ* proximity ligation assay (PLA). Consistent with the role of nuclear CRIF1 as a tumor suppressor, CRIF1 silencing promotes leukemic T cell survival in the absence of growth factors. This protective effect can be recapitulated by endogenous Lck or reconstituted Lck in leukemic T cells. All together, our results support a novel function of nuclear Lck in promoting human leukemic T cell survival through interaction with a tumor suppressor. It has important implications in defining a paradigm shift of non-canonical protein tyrosine kinase signaling.

## Introduction

Protein tyrosine kinases (PTKs) are a family of enzymes regulating diverse cellular functions that are associated with cancer hallmarks, such as cell survival (1). Many PTKs are plasma membrane-associated signal transducers that transmit signals into the nucleus in response to environmental cues. However, accumulating evidence indicates that PTKs also translocate to the nucleus and exert additional functions. Nuclear PTKs are known to change the stability and activity of nuclear residing proteins as well as to regulate nuclear gene expression (2). For example, epidermal growth factor receptor (EGFR) can regulate nuclear activity either by functioning as a transcription factor or by phosphorylating histone H4 (3,4). EGFR is present in the nuclei of proliferating liver cells and breast cancer cells (5,6). Nuclear EGFR binds to the *cyclin D1* promoter region and upregulates cyclin D1 expression to promote breast cancer cell cycle progression (6). In breast cancer cells, ErbB2 also interacts with and phosphorylates Cdc2 in the nucleus to confer resistance to Taxol-induced apoptosis (7). In addition to EGFR, other receptor and non-receptor PTKs have been detected in the nuclei of solid tumors (8,9). However, the role of nuclear PTKs in blood cancer is largely unknown.

Lymphocyte-specific protein tyrosine kinase (Lck) is a Src family kinase (SFK) predominantly expressed in T cells and plays a pivotal role in normal T cell development and homeostasis (10,11). The gene coding for *Lck* is localized near the chromosomal region with a high frequency of translocation (12). Overexpression and hyperactivation of Lck have been reported in both acute and chronic leukemias (13). Lck overexpression is also linked to poor clinical outcome to prednisone treatment in acute B lymphoblastic leukemia patients (14). In addition to blood malignancies, abnormally high expression and activity of Lck have been reported in solid tumors, such as colorectal and prostate cancers (15,16). Under physiological conditions, Lck is associated with plasma membrane and propagates signals initiated from the T cell receptors (17). However, immunohistochemical analysis of specimens from breast cancer patients revealed the presence of nuclear Lck (18). It suggests that nuclear localization of Lck may also be associated with malignant progression of hematopoietic cells.

Our previous study demonstrated that, in mouse LSTRA leukemia, Lck upregulated the expression of the *LIM domain only 2* gene through direct binding to its promoter region (19). We further provided evidence supporting the mouse LSTRA leukemic cell line as a model for the aggressive form of human large granular lymphocyte leukemia (20). These findings led us to hypothesize that Lck may also exhibit additional functions in the nuclear compartment of human leukemic cells. In the present study, we used the well-defined human T leukemic cell line Jurkat to examine the biological outcome and underlying mechanism of Lck nuclear translocation.

## Materials and methods

### Cell lines and reagents

Human Jurkat E6.1 and Jcam 1.6 T cell lines and the mouse LSTRA T cell line were maintained as described previously (21). The Jcam 1.6 cell line transfected with an expression vector containing the wild-type Lck (Jcam/Lck) was a generous gift from Dr Steven Burakoff (Icahn School of Medicine at Mount Sinai, New York City, NY, USA). CR6-interacting factor 1 (CRIF1)-knockdown stable cell lines were generated from Jcam cells using lentiviral transduction. CRIF1 shRNA (sc-97804-V) and scrambled shRNA control (sc-108080) lentiviral particles were purchased from Santa Cruz Biotechnology (Dallas, TX, USA). After 24-h serum starvation, 10^4^ Jcam cells were harvested and resuspended in 50 *μ*l of freshly thawed virus mixture (2×10^5^ infectious units). After a 6-h incubation, 500 *μ*l of complete RPMI-1640 was added. After 1 day of recovery, puromycin was added to a final concentration of 14 *μ*g/ml and cells were selected for 1 week.

### Mouse splenocyte isolation

Splenocytes were isolated from BALB/c mouse spleens as described previously (20). Briefly, the spleen was dissociated by gently pressing it through a 70-*μ*m cell strainer with a syringe plunger. Red blood cells were lysed by resuspending the cells in ammonium-chloride-potassium (ACK) lysis buffer at room temperature for 5 min. Splenocytes were then washed with cold RPMI-1640 and kept on ice for further analysis. The use of animals was approved by the Institutional Animal Care and Use Committee (IACUC) of the university.

### Pervanadate activation and dasatinib treatment

Mouse splenocytes and human T cell lines were stimulated with freshly prepared pervanadate at the final concentration of 100 *μ*M at 37°C for 5 min as described previously (22). Dasatinib was purchased from LC Laboratories (Woburn, MA, USA) and kept at −20°C as a 10-mM stock solution in DMSO. For dasatinib treatment, 10^7^ Jurkat cells were treated with DMSO as control or 500 nM of dasatinib at 37°C for 2 h. The reactions were stopped by quick cool down on ice. Pervanadate, dasatinib and DMSO control were then removed by washing cells in ice-cold phosphate-buffered saline.

### Subcellular fractionation

Cells were lysed by swelling in hypotonic buffer followed by passing through a 27-gauge needle. Light microscopy was used to ensure cell rupture before proceeding to the next step. The nuclear fraction was collected by differential centrifugation as described previously (19). Fraction purity was verified by immunoblotting of specific markers.

### Immunoprecipitation and immunoblotting

Whole cell and nuclear lysates were prepared by solubilizing whole cell and nuclear pellets in RIPA buffer, respectively. For co-immuno-precipitation experiments, proteins were extracted from the nuclear pellets using high-salt buffer (19). Target proteins were either immunoprecipitated or directly detected from total lysates after SDS-PAGE using specific antibodies according to manufacturers’ instructions. Antibodies specific for Lck, CRIF1 and epidermal growth factor receptor substrate 15 (Eps15) were purchased from Santa Cruz Biotechnology. Antibodies specific for phospho-Src family (Tyr416) and glyceraldehyde-3-phosphate dehydrogenase (GAPDH) were purchased from Cell Signaling Technology (Danvers, MA, USA). Anti-lamin B1 antibody was purchased from Abcam (Cambridge, MA, USA). Appropriate secondary antibodies conjugated with horseradish peroxidase were used to detect signals by enhanced chemiluminescence system. For signal quantitation, the bands were digitalized and analyzed by ImageJ software.

### Confocal immunofluorescence microscopy

The cells were adhered to 10-well slides, fixed, and permeabilized as previously described (21). The cells were blocked with Image-iT FX Signal Enhancer (Life Technologies, Grand Island, NY, USA) for 15 min at room temperature, and then either singly or doubly stained with primary antibodies. Subsequent labeling with Alexa Fluor-conjugated secondary antibodies and DAPI counterstain (Life Technologies) were performed to visualize primary antibodies and nuclei, respectively. Stained cells were viewed using the Olympus FV10i fluorescence confocal microscope. Images were analyzed using FluoView software (Olympus, Melville, NY, USA).

### In situ proximity ligation assay (PLA) microscopy

PLA was performed using the DuoLink PLA kit (Sigma-Aldrich, St. Louis, MO, USA) according to the manufacturer’s instructions. Briefly, 10^4^ cells were seeded on each well of 10-well slides. The adhered cells were fixed with 4% paraformaldehyde and then permeabilized with 0.2% Triton X-100. After treatment with DuoLink blocking buffer, the cells were incubated with diluted primary antibodies for Lck and CRIF1. After washing, the cells were incubated with species-specific PLA probes and two additional oligonucleotides under hybridization conditions. Hybridization occurs when PLA probes are in close proximity, which can be subsequently ligated to form a closed circle. A rolling-circle amplification step follows with polymerase to generate a concatemeric product, which can be visualized with fluorephore-labeled oligonucleotides after hybridization. The slides were counterstained with DAPI and analyzed by fluorescence microscope.

### Transient transfection

Approximately 10^4^ HEK293T cells were plated in a 10-cm dish in 10 ml of DMEM supplemented with 10% fetal bovine serum. Cells under 70% confluency were transiently transfected with 18 *μ*g of plasmids using calcium phosphate precipitation protocol (23). The cells were harvested between 42–46 h after transfection. Wild-type Lck construct as well as constitutively active (Y505F), kinase dead (Y505F/K273R), and basal kinase (Y505F/Y394F) Lck mutant constructs have been described previously (23).

### Cell number and viability

Human T cell lines were washed in plain RPMI-1640 and then incubated in serum-free RPMI-1640 at 37°C with 5% CO_2_ for 48 h. Cell viability was determined by incubation of a small fraction of cells in 0.1% trypan blue dye. Both viable and dead cells were counted with a hemocytometer under light microscope to calculate the percentage of dead cells.

### Statistical analysis

Data are presented as mean ± SE from at least three independent experiments. The significance of differences was analyzed using a Student’s t-test (SigmaPlot 11; SPSS Inc., Chicago, IL, USA). Differences were considered significant at P<0.05.

## Results

### Lck is present in the nuclear compartment of leukemia cells

**Figure 1:**
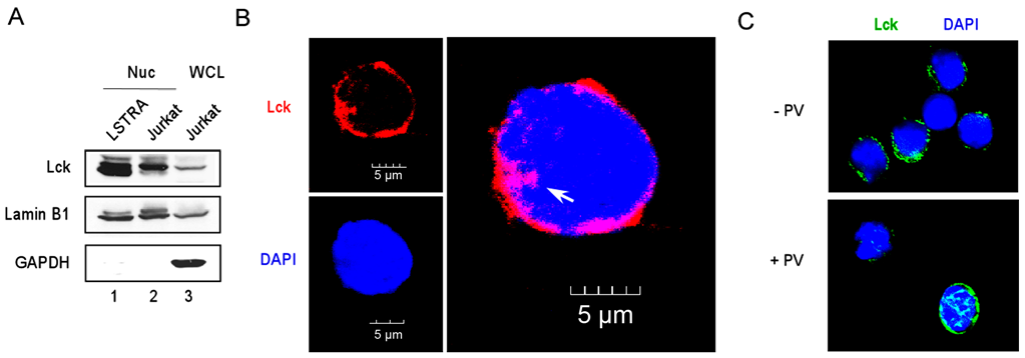
Nuclear localization of endogenous Lck in T cell lines and primary cells. (A) Nuclear (Nuc) fractions isolated from Jurkat and LSTRA cell lines were analyzed by Lck immunoblotting. GAPDH (cytosolic marker) and lamin B1 (nuclear marker) immunoblotting were also performed to verify the purity of the nuclear fraction. Jurkat whole cell lysate (WCL) was used as a positive control for markers (lane 3). (B) Jurkat cells were subjected to immunofluorescence microscopy using an anti-Lck antibody (red). Nuclei were counterstained with DAPI (blue). Nuclear staining of Lck is indicated by an arrow in the enlarged merged image. Scale bars of 5 *μ*m are shown at the bottom of the microscopy images. (C) Mouse splenocytes stimulated with pervanadate for 5 min (+PV) or left unstimulated (−PV) were analyzed by anti-Lck immunofluorescence microscopy (green) with DAPI counterstain (blue). Lck, lymphocyte-specific protein tyrosine kinase; GAPDH, glyceraldehyde-3-phosphate dehydrogenase.

LSTRA is a mouse leukemic cell line that overexpresses active Lck kinase (24). We previously showed that a large percentage of endogenous Lck was accumulated in LSTRA nuclei (Fig. 1A, lane 1) (19). To determine whether Lck trans-locates to the nuclei in primary cells, we examined mouse splenocytes by immunofluorescence microscopy using an anti-Lck antibody. As shown in Fig. 1C, Lck staining was mostly cytoplasmic in the resting cells (upper panel). To maximally activate Lck tyrosine phosphorylation, we stimulated mouse splenocytes with pervanadate, which is a potent tyrosine phosphatase inhibitor. As shown in Fig. 1C, a significant amount of Lck translocated to the nucleus of the pervanadate-stimulated cells (lower panel). This result confirms the presence of nuclear Lck in primary cells and further suggests the involvement of Lck phosphorylation in its nuclear translocation.

The Jurkat cell line was isolated from an acute T lympho-blastic leukemia patient and is one of the best characterized human leukemic T cell lines (25). We examined the presence of nuclear Lck by subcellular fractionation of Jurkat cells to isolate nuclear proteins (Fig. 1A, lane 2). Subsequent immunoblotting detected the presence of nuclear Lck (top panel) in the absence of cytoplasmic contamination (bottom panel). Confocal microscopy after staining with the anti-Lck antibody further confirmed localization of endogenous Lck in the Jurkat nucleus (Fig. 1B). These findings support a potential role of nuclear Lck in both mouse and human T cell leukemias.

### Kinase activity enhances Lck nuclear translocation

**Figure 2:**
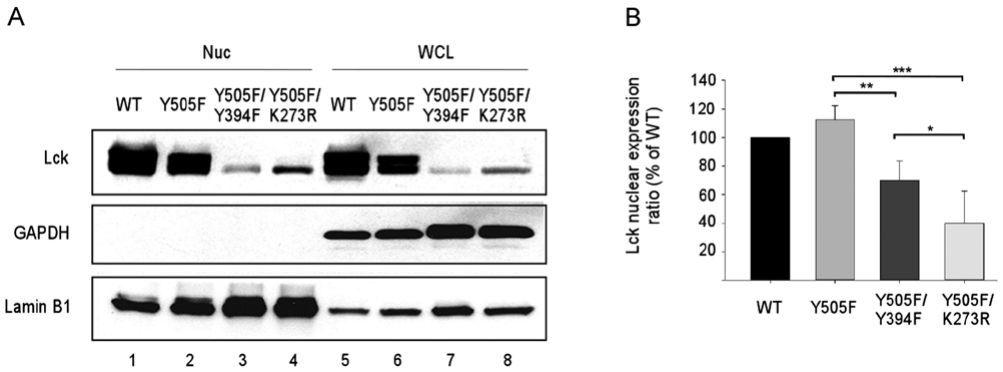
Contribution of exogenous Lck kinase activity in its nuclear translocation. (A) Nuclear fractions were isolated from HEK293T cells transfected with wild-type (WT) and three different mutant Lck constructs (lanes 1–4). Equal amount of proteins were analyzed by Lck immunoblotting (top panel). Whole cell lysates (WCL) prepared from 10% of the transfected cells were included to determine the expression levels of exogenous Lck proteins (lanes 5–8, top panel). Fraction purity was verified by GAPDH and lamin B1 immunoblotting. (B) Expression levels of nuclear Lck and total Lck were quantitated in transfected HEK293T cells to obtain the ratios of nuclear expression. For each set of transfection experiments, the ratio of nuclear wild-type Lck was set as 100%. Statistical analysis was performed on three independent transfection experiments, ^*^P<0.05, ^**^P<0.01, ^***^P<0.001. Lck, lymphocyte-specific protein tyrosine kinase; GAPDH, glyceraldehyde-3-phosphate dehydrogenase.

Similar to other SFKs, Lck activity is tightly regulated by phosphorylation of two key regulatory tyrosine residues (26). Phosphorylation of the negative regulatory Y505 results in a closed conformation and reduced Lck kinase activity. On the other hand, phosphorylation of the positive regulatory Y394 confers a fully active Lck kinase with an open conformation. To establish a correlation between Lck kinase activity and its nuclear translocation, we analyzed wild-type and three mutant Lck proteins. The Y505F mutant is locked in an open conformation and becomes constitutively active. Additional mutation on Y394 (Y505F/Y394F) reduces the kinase activity; while mutation on the key residue in kinase domain (K273R) abolishes the kinase activity. All four constructs were transfected into HEK293T cells that lack endogenous Lck. Lck immunoblotting of nuclear versus total proteins (Fig. 2A) and subsequent quantitation (Fig. 2B) showed a step-wise reduction in nuclear Lck accumulation with decreasing kinase activity.

**Figure 3:**
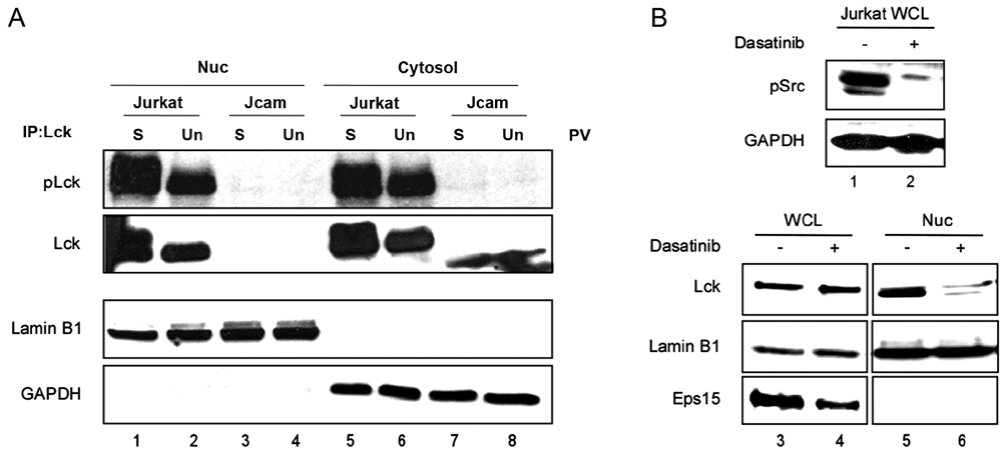
Contribution of endogenous Lck kinase activity in its nuclear translocation. (A) Nuclear and cytosolic fractions were isolated from Jurkat and Jcam cells either unstimulated (Un) or stimulated with pervanadate for 5 min (S). Equal amount of proteins from each set of lysates was immunoprecipitated (IP) with an anti-Lck antibody, and then subjected to immunoblotting with an anti-phospho-Src family (Y416) antibody to visualize Lck phosphorylation (pLck). The amount of total Lck was determined by sequential blotting with the anti-Lck antibody. A fraction of the total lysates was probed for GAPDH and lamin B1 to verify fraction purity. (B) Jurkat cells were treated with dasatinib or vehicle control for 2 h. Whole cell lysates (WCL) were prepared from a fraction of the cells and analyzed by immunoblotting using antibodies specific for phospho-Y416-Src family (pSrc) and GAPDH (lanes 1 and 2). Nuclear proteins isolated from the remaining cells were subjected to immunoblotting using antibodies specific for Lck, lamin B1 and Eps15 (cytosolic marker) (lanes 5 and 6). Equal amount of whole cell lysates was also analyzed by immunoblotting using antibodies specific for Lck, lamin B1 and Eps15 (lanes 3 and 4). Lck, lymphocyte-specific protein tyrosine kinase; GAPDH, glyceraldehyde-3-phosphate dehydrogenase; Eps15, epidermal growth factor receptor substrate 15.

To further establish the role of Lck kinase activity in its nuclear translocation in human T cells, we examined both Jurkat and its Lck-deficient derivative Jcam cell lines. In the Jcam cells, Lck is inactive due to truncation and is expressed at a very low level (27). Phosphorylation of the positive regulatory Y394 is indicative of Lck kinase activity. We determined the level of Y394 phosphorylation by Lck immunoprecipitation and subsequent immunoblotting using an antibody that specifically recognizes phosphorylation of this conserved tyrosine residue in all SFKs. As shown in Fig. 3A, in the Jcam cells, the truncated kinase-dead Lck (lanes 7 and 8) could not translocate to the nucleus either without (lane 4) or with (lane 3) pervanadate stimulation. On the other hand, pervanadate stimulation of Jurkat cells greatly enhanced Y394 phosphorylation and nuclear translocation of Lck (Fig. 3A, lanes 1 and 2). This was consistent with pervanadate-induced Lck translocation into the nuclei of primary cells (Fig. 1C). As an independent confirmation, we treated Jurkat cells with an SFK inhibitor, dasatinib. As shown in Fig. 3B, in the absence of SFK activity (compare lanes 1 and 2), the amount of nuclear Lck was greatly diminished (compare lanes 5 and 6) in the Jurkat cells.

### Lck interacts with CRIF1

**Figure 4:**
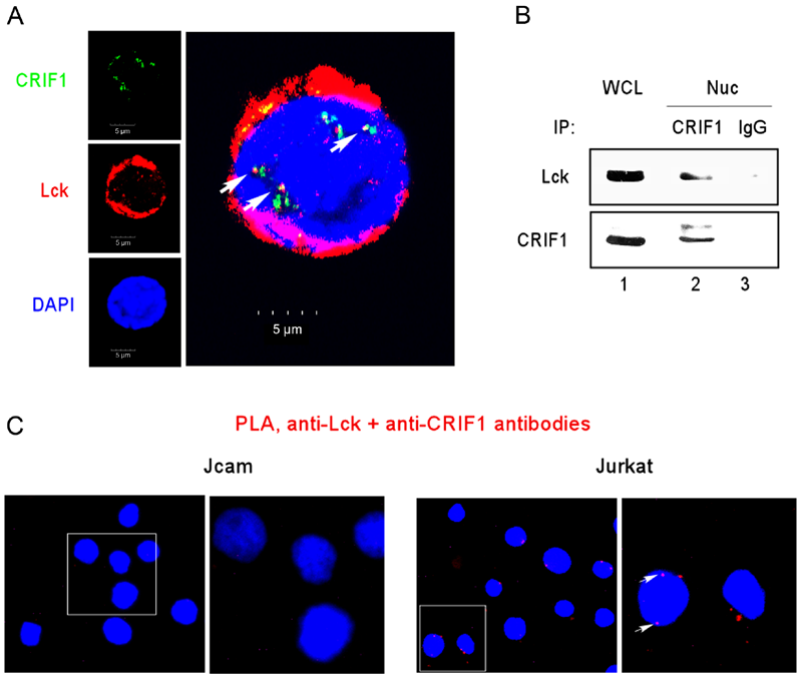
Lck interacts with CRIF1. (A) Jurkat cells were subjected to immunofluorescence microscopy with two-color staining for CRIF1 (green) and Lck (red). Cells were also counterstained with DAPI to visualize nuclei (blue). White arrows in the enlarged three-color merged image indicate co-localization of Lck and CRIF1 in the nucleus (white dots). (B) Nuclear proteins isolated from Jurkat cells were immunoprecipitated with either an anti-CRIF1 antibody (lane 2) or control IgG (lane 3), and then subjected to Lck and CRIF1 immunoblotting. A fraction of Jurkat whole cell lysate was loaded as positive controls (lane 1). (C) Jurkat and Jcam cells were subjected to PLA microscopy using primary antibodies specific for Lck and CRIF1. The areas bordered by white lines are enlarged on the right for enhanced visualization. Red fluorescence indicates Lck and CRIF1 interaction *in situ*. White arrows denote Lck and CRIF1 interaction in the nucleus. Lck, lymphocyte-specific protein tyrosine kinase; CRIF1, CR6-interacting factor 1; PLA, proximity ligation assay.

We previously showed that Lck may function as a transcription factor by binding to specific target genes (19). To identify additional Lck-interacting partners, we performed mass spectrometry on Lck immunoprecipitates prepared from LSTRA lysates. Our previous proteomic analysis identified CRIF1 as one of the top candidates (unpublished data). To confirm the interaction between Lck and CRIF1 in human leukemic T cells, we performed immunofluorescence microscopy and detected the co-localization of Lck and CRIF1 in the Jurkat nucleus (Fig. 4A). Co-immunoprecipitation between Lck and CRIF1 in nuclear extracts prepared from Jurkat cells also confirmed the association of Lck and CRIF1 (Fig. 4B, lane 2).

To further validate the close-range interaction between Lck and CRIF1, we performed *in situ* PLA microscopy. A positive PLA result relies on two molecules in the proximity of 16 nm or below, which reflects true protein-protein interaction. As shown in Fig. 4C, a PLA signal was detected in the Jurkat nucleus (right panels). Additional PLA staining was observed outside the nuclei of Jurkat cells (Fig. 4C, right panels). This is consistent with our earlier observation of Lck interaction with CRIF1 in mitochondria (unpublished data). As a negative control, no PLA signal was detected in the Lck-deficient Jcam cells (Fig. 4C, left panels). All together, these results support a close interaction between Lck and CRIF1 in the nuclear compartment of Jurkat cells.

### Lck promotes leukemic T cell survival by inhibiting CRIF1 function

**Figure 5:**
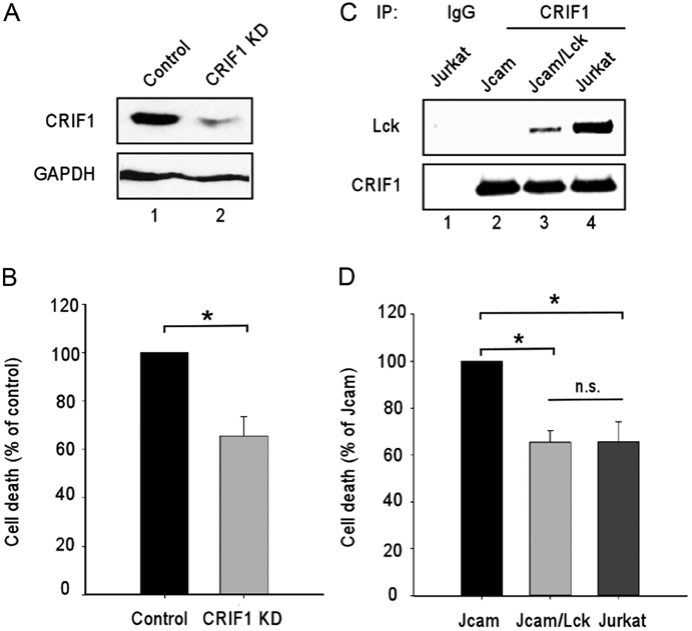
CRIF1 knockdown and association with Lck confer resistance to cell death induced by serum deprivation. (A) Whole cell lysates prepared from Jcam cells stably transduced with sh-control and sh-CRIF1 (CRIF1 KD) were subjected to CRIF1 and GAPDH immunoblotting. (B) Cell death after serum deprivation was measured in transduced Jcam cells and presented as a percentage in comparison to the sh-control Jcam cells. (C) Whole cell lysates were prepared from Jurkat, Jacm, and Jcam reconstituted with wild-type Lck (Jcam/Lck). Normalized proteins were immunoprecipitated with an anti-CRIF1 antibody (lanes 2–4) or control IgG (lane 1), and then subjected to sequential blotting with anti-Lck and anti-CRIF1 antibodies. (D) Cell death after serum deprivation was measured in Jurkat, Jcam and Lck-expressing Jcam cells by trypan blue exclusion assay. The ratio of cell death in the Jcam cells was set as 100% in each set of experiment. Statistical analysis was performed using data from three independent experiments, ^*^P<0.05 or non-significant (n.s.). Lck, lymphocyte-specific protein tyrosine kinase; CRIF1, CR6-interacting factor 1; GAPDH, glyceraldehyde-3-phosphate dehydrogenase.

Nuclear CRIF1 is known as a tumor suppressor by blocking cell cycle progression and reducing cell survival. The tumor-suppressing activities of CRIF1 are largely mediated by its interaction with distinct nuclear proteins, such as cyclin-dependent kinase 2 (CDK2) and Nur77 (28,29). To determine the role of CRIF1 in human leukemic T cell survival in the absence of Lck, we knocked down CRIF1 expression in Jcam cells by RNA interference (Fig. 5A, lane 2). Both the control and CRIF1-knockdown Jcam cells were subjected to serum starvation to compare their sensitivity to growth factor deprivation. Consistent with the role of CRIF1 as a tumor suppressor, the CRIF1-knockdown Jcam cells were more resistant to starvation-induced cell death (Fig. 5B).

Since Lck binds to CRIF1 in the nucleus (Fig. 4), we hypothesized that Lck interaction with CRIF1 may interfere with CRIF1’s function as a tumor suppressor. Indeed, similar to Jcam cells with CRIF1 knockdown (Fig. 5B), Jurkat cells were more resistant to starvation-induced cell death as compared to Jcam cells (Fig. 5D). To verify the contribution of Lck, wild-type Lck was reconstituted in Jcam cells and was capable of binding to CRIF1 (Fig. 5C, lane 3). The exogenous Lck confers protection from starvation-induced cell death to a level similar to endogenous Lck in Jurkat (Fig. 5D). These results support the role of Lck in promoting cell survival by its interaction with CRIF1 and, potentially, inhibition of CRIF1 activity.

## Discussion

In summary, our present study revealed a novel function of nuclear Lck in promoting cell survival through interaction with a tumor suppressor. However, we do not exclude the possibility that nuclear Lck may exert additional functions. For example, our previous study demonstrated the role of Lck as a nuclear transcription factor in regulating genes important in oncogenesis (19). Other PTKs, such as EGFR, also exhibit multiple functions in the nucleus. Nuclear EGFR binds to the promoter regions of distinct target genes, upregulates gene expression, and promotes cell cycle progression (6,30). Interaction of EGFR with DNA protein kinase in the nucleus, on the other hand, enhances DNA damage repair and augments breast cancer cell’s resistance to cisplatin and ionizing radiation treatment (31). Additionally, modulation of PCNA and Cdc2 stability and activity through their association with nuclear EGFR contributes to uncontrolled proliferation and DNA repair in breast cancer cells (7,32). Our data further expand the biological significance of nuclear PTKs from receptor PTK to non-receptor PTK as well as from solid tumors to blood cancer.

Our results also support a positive effect of Lck kinase activity on its nuclear translocation. Consistent with our findings, kinase activity of EGFR is also important in its nuclear translocation in breast cancer cells (33). It should be noted, however, that a small amount of kinase-dead Lck (Fig. 2B) and inactive Lck (Fig. 3B) can still be detected in the nuclear compartment. It is possible that other kinase-independent mechanisms also contribute to Lck nuclear translocation. While a nuclear localization signal (NLS) has been identified in EGFR (34), there is no discernible NLS in Lck. It remains to be determined whether and how a non-classical NLS may mediate nuclear trafficking of Lck.

Our mass spectrometry analysis identified CRIF1 as a novel Lck-interacting partner. Consistent with its role as a tumor suppressor, CRIF1 interacts with and inhibits CDK2 to induce cell cycle arrest in leukemia cells (28). CRIF1-induced cell cycle arrest can also be mediated by its interaction with other nuclear proteins, including GADD45 and Nur77 (29,35). Our CRIF1 knockdown data in Jcam cells further support the role of CRIF1 in sensitizing leukemic cells to cell death, another important cancer hallmark (36). CRIF1 interaction with nuclear proteins may also contribute to its role in cell death. More importantly, our data suggest that Lck can bind to CRIF1 and inhibit its function as a tumor suppressor (Fig. 5). It remains to be determined whether Lck competes with other nuclear proteins in binding to CRIF1.

Other than an important nuclear regulator, CRIF also plays a critical role in the mitochondria (37). CRIF1 associates with the inner membrane of mitochondria and participates in the synthesis of oxidative phosphorylation polypeptides encoded by the mitochondrial circular genome. CRIF1 also facilitates their insertion into the inner membrane for the assembly of functional electron transport chain complex. As expected, CRIF1 deficiency in the mitochondrial compartment results in mitochondrial dysfunction. In brain-specific *CRIF1*-knockout mice, fatal neurodegeneration is associated with abnormal mitochondrial morphology (37). A recent study on an Alzheimer’s disease animal model further suggests a protective role of mitochondrial CRIF1 in neuronal cells against apoptosis (38). While the functions of nuclear CRIF1 are not fully addressed in neuronal cells, these studies do suggest that CRIF1 can promote the survival of distinct cell types, such as neuronal cells. In our T cell model, CRIF1 knockdown in Jcam cells also resulted in slightly more dead cells before serum deprivation (data not shown). However, under stress condition, CRIF1 promoted Jcam cell death (Fig. 5B). The overall effect of CRIF1 on cell survival, therefore, may depend on the cell type and the experimental condition.

Consistent with the dual functions of CRIF1 in different organelles, we also observed mitochondrial localization of CRIF1 in Jurkat leukemia (unpublished data). Similarly, we confirmed the presence of mitochondrial Lck in Jurkat T cells. The concomitant localization of Lck in the cytoplasm, mitochondria and nucleus further highlights the complexity of crosstalk between different subcellular compartments. Other receptor and non-receptor PTKs have been reported to exhibit diverse functions in the cytoplasm, mitochondria and nucleus (39–41). These findings of non-canonical signaling represent an important paradigm shift of how PTKs coordinately regulate cellular activities. They also provide critical insights in identifying novel molecular targets in interfering with the oncogenic signal transduction network.

## Abbrevations

PTK: protein tyrosine kinase
EGFR: epidermal growth factor receptor
SFK: Src family kinase
CRIF1: CR6-interacting factor 1
Eps15: epidermal growth factor receptor substrate 15
GAPDH: glyceraldehyde-3-phosphate dehydrogenase
PLA: proximity ligation assay
CDK2: cyclin-dependent kinase 2
NLS: nuclear localization signal
Lck: lymphocyte-specific protein tyrosine kinase
